# Pan-cancer analysis and single-cell analysis reveals FAM110B as a potential target for survival and immunotherapy

**DOI:** 10.3389/fmolb.2024.1424104

**Published:** 2024-08-07

**Authors:** Yuwei Li, Xiaoxi Li, Bihua Wu, Shuangyan Su, Yunpeng Su, Le Guo

**Affiliations:** ^1^ Department of Medical Microbiology and Immunology, School of Basic Medical Sciences, Dali University, Dali, Yunnan, China; ^2^ Department of General Surgery, School of Clinical Medicine, Dali University, Dali, Yunnan, China

**Keywords:** FAM110B, pan-cancer, prognosis, tumor microenvironment, targeted therapy, bioinformatic analysis, immunotherapy

## Abstract

**Background:** FAM110B belongs to the family that has a 110 sequence similarity (FAM110) and is located in the centrosome and mitotic spindle. FAM110B has been linked to tumor cell growth in earlier research. Uncertainty exists regarding FAM110B’s function within the tumor microenvironment is unclear as well as pan-cancer.

**Methods:** In order to assess the variation in FAM110B expression within normal and pan-cancer tissues, we combined the TCGA and GTEx databases. The cBioPortal database and the GSCALite platform were used to examine the variation in genome and methylation alteration of FAM110B. Cox regression, Kaplan-Meier, and SangerBox were employed to examine the clinical features and prognosis of FAM110B and pan-cancer. The purpose of the correlational research was to investigate the associations within immunerelated genes, tumor mutation burden, microsatellite instability, immune-related genes, and immunological checkpoints and FAM110B expression. ESTIMATE, EPIC, QUANTISEQ, and MCPCOUNTER methods were used to calculate the interaction among FAM110B expression as well as the tumor immune microenvironment. The immunoinfiltration and function of FAM110B were analyzed by single-cell databases (TISCH and CancerSEA). Finally, we evaluated the sensitivity of FAM110B to small-molecule medications through GDSC and CTRP databases.

**Results:** The transcription and protein expression of FAM110B varies significantly throughout cancer types, and this has predictive value for the prognosis of some tumors; including brain lower grade glioma (LGG), stomach adenocarcinoma (STAD), pancreatic adenocarcinoma (PAAD), etc. In the tumor microenvironment, the expression level of FAM110B was associated with immune cell infiltration, immune checkpoint immune regulatory genes, tumor mutational burden, and microsatellite fragility to a certain extent.

**Conclusion:** This work investigates the possibility of utility of FAM110B as a marker to forecast pan-cancer immunotherapy response, providing a theoretical basis for cancer therapy.

## 1 Introduction

The unchecked growth of aberrant cells and aberrant immune system recognition are the hallmarks of cancer, the second most prevalent cause of death worldwide. Over 10 million people will lose their lives to cancer by 2020, with 5.47 million of those deaths occurring in China, 2.28 million in the United States, and 1.32 million in India. There will be 19.3 million new instances of cancer globally (Sung et al., 2021). According to Cancer Statistics, there will be over 2 million new instances of cancer in the United States in 2024 (Siegel et al., 2024). In recent years, due to the long-term inadequate therapeutic efficacy of traditional strategies, some newly developed immunotherapies have received more and more attention (Yang et al., 2014), such as checkpoint inhibitors and immune cell therapy (Bruni et al., 2020). However, the particular tumor antigen plays a major role in the specificity of immunotherapy (Li and Ding, 2020). Genomic mutations and epigenetic changes in tumor antigens render immunotherapy ineffective for tumor proliferation (Balon et al., 2022; Hanahan, 2022). Therefore, there is a pressing need to look into more effective immunotherapy-related tumor prognostic markers.

The family with sequence similarity 110 (FAM110), consists of the following members: FAM110A, FAM110B, and FAM110C, is located in the centrosome and mitotic spindle, and its family members influence different cell cycle processes (Hauge et al., 2007). In studies of cell cycle regulation transcripts, FAM110A expression peaked in G2 phase and showed an expression profile similar to those of several well-characterized genes involved in surveillance of mitotic processes. The ectopic expression of FAM110B and FAM110C can affect the cell cycle progression in G1 phase. Certain FAM gene family members have emerged as intriguing targets for therapy as well as indicators of prognosis for the treatment of different kinds of cancer in recent years. For instance, Tetraspanin1 stimulates FAM110A’s enhanced expression to encourage the growth and spread of pancreatic cancer (PAAD) cells (Huang et al., 2022); overexpression of FAM110B can inhibit Wnt/β-catenin signaling and thus inhibit the malignant biological activity of non-small cell lung cancer (NSCLC) cells (Xie et al., 2020); FAM110C contributes to the growth and migration of glioma (GBM) with isocitrate dehydrogenase1 (IDH1) mutation (Ren et al., 2023). FAM110B was the purported progenitor of the whole FAM110 family among these individuals. However, the published literature on FAM110B is extremely limited, especially on cancer. Despite the fact that research indicates FAM110B regulates breast cancer (Cava et al., 2021), NSCLC (Xie et al., 2020), and uterine corpus endometrial carcinoma (UCEC) (Chi et al., 2023), studies on the connections between FAM110B expression and tumor immune cell invasion, tumor mutation burden (TMB), microsatellite instability (MSI), drug sensitivity, and the mechanism of action with pan-cancer have not been clarified. Only FAM110B was found to be a potential immune-related marker for pancreatic cancer in our earlier research (Li et al., 2023). Thus, a comprehensive analysis of the pan-cancer function of FAM110B is necessary.

In order to elucidate the expression, aberrant variation, and clinical importance of FAM110B in pan-cancer, a thorough bioinformatics evaluation of the protein was carried out in the present investigation using several databases. Further analysis was done on FAM110B’s function in the tumor immunological milieu, and the connection between FAM110B, immunotherapy response, and associated sensitive medications was assessed. These results not only suggest that FAM110B may be an effective prognostic biomarker closely associated with tumor immunomodulatory mechanisms and anti-tumor drug resistance, but also reveal its role as a potential predictor of pan-cancer immunotherapy.

## 2 Materials and methods

### 2.1 Acquisition of pan-cancer expression data

A standardized pan-cancer dataset was downloaded from the UCSC (https://xenabrowser.net/, accessed on 28 December 2023) database: the TCGA, TARGET, and GTEx (PANCAN, N = 19131, G = 60499) (See Supplementary Material for detailed procedures). The expression difference between normal and tumor samples in each tumor was calculated using R software (version 3.6.4), and the difference significance was analyzed using unpaired Wilcoxon Rank Sum and Signed Rank Tests. FAM110B gene differential expression analysis of unpaired normal and cancer tissues, clinical feature correlation analysis, survival analysis, and immune feature correlation analysis were performed by SangerBox3.0 (http://sangerbox.com/, accessed on 28 December 2023) (Shen et al., 2022). In Table 1, we report the abbreviation for each tumor type.

**TABLE 1:** Tumor types and abbreviations.

Abbreviation	Full name
ACC	Adrenocortical carcinoma
ALL	Acute lymphoblastic leukemia
AML	Acute myeloid leukemia
AST	Astrocytoma
BLCA	Bladder urothelial carcinoma
BRCA	Breast invasive carcinoma
CESC	Cervical squamous cell carcinoma and endocervical adenocarcinoma
CHOL	Cholangiocarcinoma
CML	Chronic myelogenous leukemia
COAD	Colon adenocarcinoma
COADREAD	Colon adenocarcinoma/Rectum adenocarcinoma Esophageal carcinoma
DLBC	Diffuse large b-cell lymphoma
ESCA	Esophageal carcinoma
GBM	Glioblastoma multiforme
GBMLGG	Glioma
HNSC/HNSCC	Head and neck squamous cell carcinoma
KICH	Kidney chromophobe
KIPAN	Pan-kidney cohort (KICH + KIRC + KIRP)
KIRC	Kidney renal clear cell carcinoma
KIRP	Kidney renal papillary cell carcinoma
LAML	Acute myeloid leukemia
LGG	Brain lower grade glioma
LIHC	Liver hepatocellular carcinoma
LUAD	Lung adenocarcinoma
LUSC	Lung squamous cell carcinoma
MEL	Melanoma
NSCLC	Non-small cell lung cancer
ODG	Oligodendroglioma
OV	Ovarian serous cystadenocarcinoma
PAAD	Pancreatic adenocarcinoma
PCPG	Pheochromocytoma and Paraganglioma
PRAD	Prostate adenocarcinoma
RB	Retinoblastoma
RCC	Renal cell carcinoma
READ	Rectum adenocarcinoma
SARC	Sarcoma
SKCM	Skin cutaneous melanoma
STAD	Stomach adenocarcinoma
STES	Stomach and esophageal carcinoma
TGCT	Testicular germ cell tumors
THCA	Thyroid carcinoma
THYM	Thymoma
UCEC	Uterine corpus endometrial carcinoma
UCS	Uterine carcinosarcoma
UM	Uveal melanoma
UVM	Uveal melanoma

### 2.2 FAM110B protein expression and subcellular localization

The Human Protein Atlas (HPA, https://www.proteinatlas.org/, accessed on 28 December 2023) database was used to explore the protein expression levels of FAM110B in different cancer patient tissues. Additionally, the subcellular localization of the FAM110B gene was determined using the human gene database Genecards (https://www.genecards.org/, accessed on 26 December 2023).

### 2.3 Prognostic analysis of FAM110B

We selected the overall survival (OS), disease-specific survival (DSS), disease-free interval (DFI), and progression-free interval (PFI) of 33 cancer patients to analyze the relationship between FAM110B expression and prognosis according to reference (Liu J. et al., 2018). A Cox proportional hazards regression model was established using the coxph function of the R package survival (version 3.2-7) to analyze the relationship between gene expression and prognosis in each tumor, using log-rank test, statistical tests were performed to obtain prognostic significance. We used the R software package “maxstat” to calculate the optimal truncation value of FAM110B. Based on this, the patients were divided into high and low groups, and the survfit function of the R software package “survival” was further used to analyze the prognostic difference between the two groups. Kaplan-Meier curve showed the relationship between FAM110B expression and OS in patients.

### 2.4 Genetic alteration, methylation, and RNA modification analysis

Access the cBioPortal database online (http://www.cbioportal.org/, accessed on 30 January 2024) and select the “FAM110B,” and “Cancer Types Summary” modules to obtain information on the genomic alteration types and frequencies of FAM110B in pan-cancer (Cerami et al., 2012). Gene Set Cancer Analysis (GSCA, http://bioinfo.life.hust.edu.cn/GSCA/, accessed on 30 January 2024) to obtain FAM110B gene copy number variation (CNV) and methylation modification, based on Spearman correlation analysis, FAM110B expression of CNV and DNA methylation (Liu C. J. et al., 2018). Finally, RNA modification analysis of 44 methylation regulators involved in n1-methyladenosine (m1A) and 5-methylcytosine (m5C) n6-methyladenosine (m6A) addition in pan-cancer tissues across TCGA was performed using SangerBox3.0.

### 2.5 Single-cell functional analysis of FAM110B

The Cancer single-cell state Atlas (CancerSEA, http://biocc.hrbmu.edu.cn/CancerSEA/, accessed on 28 December 2023) is an analytical tool that studies the function of 14 tumor-associated cells in 900 cancer cells at the single-cell level (Yuan et al., 2019). Sequencing quality was assessed by FastQC (version 0.11.2). The single-cell function of FAM110B in tumors was analyzed using this database.

### 2.6 Immune cell infiltration analysis and single-cell data sequencing validation

To conduct a reliable immune correlation assessment, we used the QUANTISEQ, MCPCOUNTER (Becht et al., 2016), and EPIC (Racle et al., 2017) algorithms to calculate the Spearman’s correlation coefficient between the FAM110B gene and immune cell infiltration in each tumor and presented the results in the form of a heat map. Online access to Tumor Immune Single-cell Hub 2 (TISCH2, http://tisch.comp-genomics.org/home/, accessed on 31 January 2024) (Li et al., 2020; Han et al., 2022) was conducted to determine the expression level of FAM110B in each cell type (see Supplementary Material).

### 2.7 Association of FAM110B expression with the tumor microenvironment (TME)

To evaluate the relationship between FAM110B expression and TME, the R software package ESTIMATE (version 1.0.13) (Yoshihara et al., 2013) was used to calculate Stromal, Immune, and ESTIMATE scores for each patient in each tumor based on gene expression. Further, Spearman’s correlation coefficient of FAM110B expression and immune infiltration score in each tumor was calculated using the corr.test function of the R package psych (version 2.1.6) and visualized with the R package “ggplot2 (3.3.6)”. The TIMER2.0 database (http://timer.comp-genomics.org/) was used to analyze the correlation of target genes with marker genes related to M1/M2 macrophages, endotheliocyte, and cancer-associated fibroblasts (CAFs) (Li et al., 2017; Li et al., 2020). Further, Spearman correlation was used to analyze the expression relationship between FAM110B and 60 immune checkpoint pathway genes (Thorsson et al., 2018) and 150 immune regulatory genes (Hu et al., 2021) (see Supplementary Material).

### 2.8 FAM110B expression and immunotherapy response

It is now widely accepted that genomic heterogeneity of tumors closely impacts the response to treatment with immune checkpoint inhibitors and patient prognosis, including TMB, MSI, and other indicators. We from GDC (https://portal.gdc.cancer.gov/, accessed on 30 January 2024) by all the TCGA MuTect2 software downloaded sample level 4 Simple Nucleotide for the variation dataset, we calculated the TMB for each tumor using the R software package Maftools (version 2.8.05) (Beroukhim et al., 2010). MSI scores for an individual tumor were obtained from the previous study by Russell Bonneville et al. (2017). The collated gene expression data were integrated with the MSI scores and purity data from the pan-cancer samples, and Spearman correlation analysis was performed. Immunotherapy response prediction and biomarker assessment of FAM110B were predicted from the TIDE website (http://tide.dfci.harvard.edu, accessed on 2 February 2024). TISIDB database (http://cis.hku.hk/TISIDB/) which is composed of many data types to evaluate the interaction between cancer and the immune system was used to analyze the relationship between FAM110B expression and molecular or immune subtypes in pan-cancer.

### 2.9 Drug sensitivity analysis

GSCALite (http://bioinfo.life.hust.edu.cn/web/GSCALite/, accessed on 30 January 2024) was used to predict the FAM110B targeted sensitive drugs, and the bubble chart displays the relationship between the drug’s half-inhibitory concentration (IC50) and FAM110B expression. Considering an adjusted *p*-value threshold of <0.05, False Discovery Rate (FDR) were calculated for each tumor CNV, methylation, and drug sensitivity (Liu C.-J. et al., 2022).

### 2.10 Statistical analysis

The bioinformatics analysis in this study was partially supported by Sangerbox. The Spearman correlation coefficient was used to evaluate associations between variables. The log-rank test was used in survival analysis. For all statistical comparisons, significance levels were set at *p* < 0.05.

## 3 Results

### 3.1 Variations in FAM110B expression and location

**FIGURE 1:**
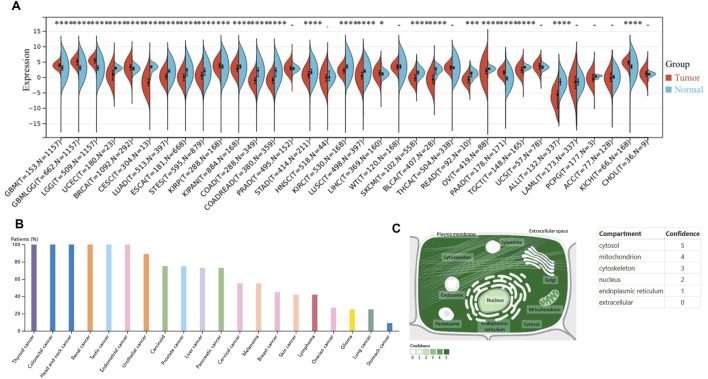
Variations in FAM110B expression between pan-cancer types. **(A)** Using the TCGA and GTEx datasets, FAM110B mRNA expression levels were determined in both normal and pan-cancer tissues. **(B)** The HPA database provided the levels of FAM110B protein expression. **(C)** The localization of FAM110B was obtained from the Genecards database. *p < 0.05, **p < 0.01, ***p < 0.001, ****p < 0.0001.

We performed an additional investigation to look into the variations in FAM110B expression between tumor and healthy tissue in pan-cancer. Through the integration of the TCGA and GTEx databases, we discovered that, in comparison with healthy tissues, FAM110B mRNA was substantially elevated in 8 tumor types (GBM, GBMLGG, LGG, BRCA, KIRP, KICH, PAAD, and LIHC; all *p* < 0.05), while it was substantially decreased in 17 kinds of cancer (KIPAN, BLCA, ALL, COAD, COADREAD, READ, CESC, STAD, TCGT, KIRC, LUAD, OV, ESCA, STES, LUSC, SKCM, and UCEC; all *p* < 0.05) (Figure 1A). Further analysis via the HPA database revealed that the expression frequency of FAM110B protein was 100% in tumor types such as thyroid cancer (4/4), colorectal cancer (12/12), head and neck cancer (4/4), renal cancer (12/12), testis cancer (10/10), and endometrial cancer (11/11) (Figure 1B). Furthermore, using the Genecards database, we discovered that the majority of FAM110B’s location was inside the cytosol (Figure 1C).

### 3.2 Copy number variance and methylation lead to the abnormal expression of FAM110B within pan-cancers

**FIGURE 2:**
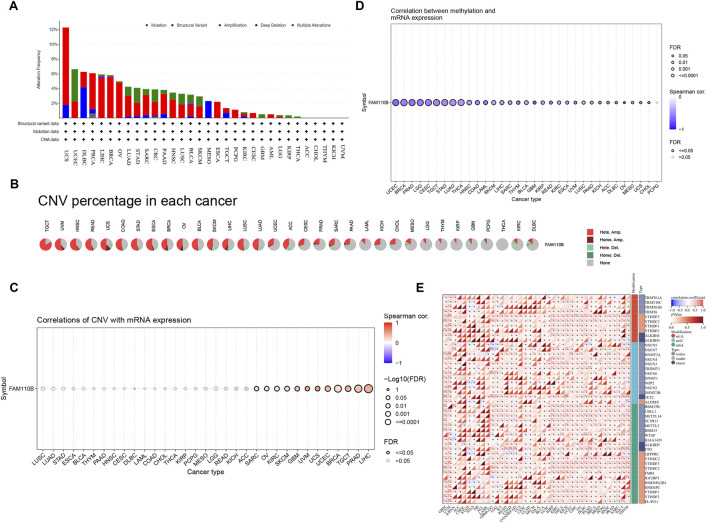
Genetic alteration and epigenetic modification of FAM110B. **(A)** Genetic alterations of FAM110B in pan-cancer using the cBioPortal. **(B)** Each cancer’s heterozygous and homozygous copy number variation (CNV) for FAM110B is displayed in a pie chart. **(C,D)** The correlation between FAM110B expression and CNV **(C)** and methylation **(D)** in pan-cancer genes. The dots’ sizes indicate the statistical signal; the bigger the dot, the higher the statistical. **(E)** Spearman correlation of FAM110B expression with m1A, m5C, and m6A regulatory gene expression. *p < 0.05.

Because malignancies showed variable expression of FAM110B, we used cBioPortal and GSCALite, two online services, to examine the genetic changes as well as epigenetic regulatory alterations of this protein. According to Figure 2A, “Amplification” was the primary genetic modification type of FAM110B, with UCS (10.53%), BRCA (5.54%), LIHC (5.38%), PRAD (4.86%), and OV (4.79%) being the most common. “Mutation” was most frequently observed within UCEC (4.35%), STAD (2.05%), CRC (1.68%), LUSC (1.44%), and SKCM (1.35%). “Deep deletion” was mainly seen in DLBC (4.17%), MESO (2.30%), and UCS (1.75%). Except for “Multiple Alterations” of PRAD (0.61%), the incidence of FAM110B gene mutations in “Structural Variation” as well as “Multiple Alterations” was typically below 0.5%. CNVs are significant abnormalities that cause changes in the expression of genes involved in the development and growth of tumors (Chen et al., 2019). The TCGA database’s CNV information was examined in order to find FAM110B variations. We discovered that FAM110B exhibited heterozygous amplification in STAD, READ, UVM, TGCT, HNSC, and ESCA, reaching up to 50% (Figure 2B). We examined the connection between FAM110B mRNA expression and CNV. The results from the GSCA database demonstrated that FAM110B expression had a strong association alongside CNV in 12 tumors; in contrast, the correlations were not significant in 21 tumors (Figure 2C), indicating that CNV could not be the sole variable that causes abnormal FAM110B expression and that the fundamental processes that result in inappropriate expression might be erratic among various tumors. It has been demonstrated that abnormal DNA methylation may hasten the growth of tumors by controlling cell division and causing senescence or apoptosis (Pfeifer, 2018). We discovered that FAM110B mRNA expression in the majority of tumor types, particularly in BRCA, UCEC, PRAD, LGG, CESC, TCGT, STAD, LUAD, and THCA, was substantially negatively linked with DNA methylation levels (Figure 2D).

Growing data indicates that RNA modification networks may be good targets for cancer treatment because they were dysregulated in human malignancies (Barbieri and Kouzarides, 2020). The connection between FAM110B expression as well as RNA modification-related genes can be seen within Figure 2E. We discovered that FAM110B expression often showed a favorable correlation with pan-cancer gene expression associated with m1A, m5C, and m6A, particularly in YTHDF3, YTHDC1, NSUN3, TET2, and METTL14. The aforementioned findings suggest that FAM110B’s aberrant expression in various cancers may be directly linked to its gene variant and involvement in epigenetic alteration.

### 3.3 Correlation of FAM110B expression with clinic pathological features and prognosis

**FIGURE 3:**
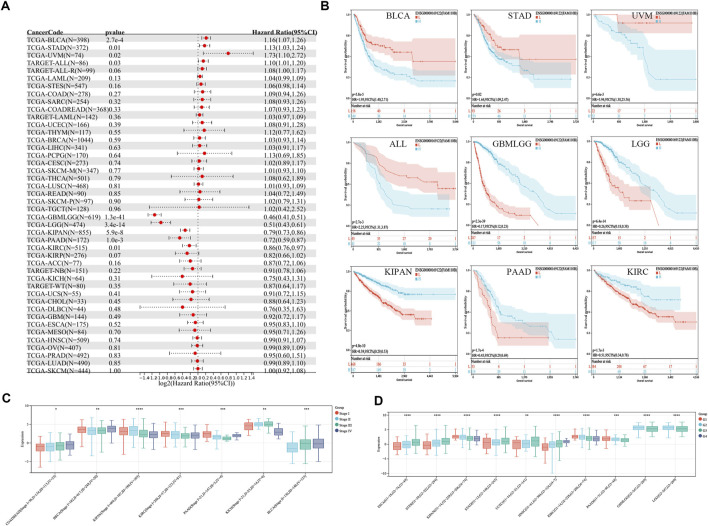
Relationship between FAM110B expression and clinical characteristics and prognosis. **(A)** The univariate regression for OS in pan-cancer. **(B)** Kaplan-Meier curves of OS with significance for nine different cancer types (BLCA, STAD, UVM, ALL, GBMLGG, LGG, KIPAN, PAAD, and KIRC), L: low expression group of FAM110B, H: high expression group of FAM110B. **(C,D)** Relationship between the expression of FAM110B and tumor clinical staging and tumor histological grading. *p < 0.05, **p < 0.01, ***p < 0.001, ****p < 0.0001.

To assess the connection between FAM110B expression as well as patient survival, we investigated the connection between FAM110B expression and OS, DSS, PFI, and along with DFI within 33 cancer types (Supplementary Figure S1). High expression of FAM110B was either protective or a risk factor for OS among individuals with various kinds of tumors, according to Cox regression model assessment (Figure 3A). According to Kaplan-Meier survival curves, those suffering from BLCA, STAD, UVM, and ALL had considerably worse prognoses when high expression of FAM110B was present; BLCA showed the strongest correlation with FAM110B. Conversely, in GBMLGG, LGG, KIPAN, PAAD, and KIRC, higher levels of FAM110B expression had a significant association with improved prognosis (Figure 3B). Seven malignancies showed an important distinction when FAM110B expression levels were further evaluated from a pan-cancer perspective at various clinical as well as pathological phases (Figure 3C). In addition, the expression of FAM110B decreased with increasing histological grade among individuals with KIPAN, KIRC, PAAD, GBMLGG, and LGG. On the other hand, individuals who had ESCA, STES, STAD, UCEC, and HNSC showed the opposite pattern (Figure 3D) (all *p* < 0.05).

### 3.4 Single-cell function evaluation of FAM110B

**FIGURE 4:**
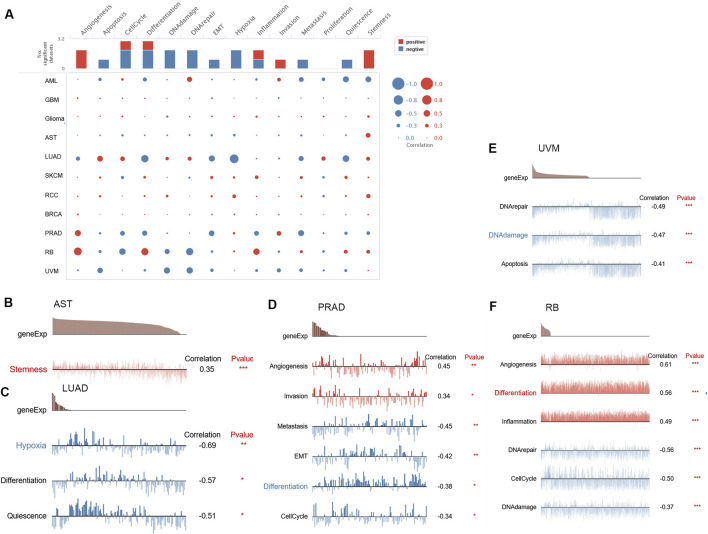
The CancerSEA database’s single-cell functional analysis reveals the role of FAM110B. **(A)** Functional status of FAM110B in different human cancers. **(B–F)** Functional status and FAM110B in AST, LUAD, PRAD, RB, and UVM were correlated. *p < 0.05, **p < 0.01, ***p < 0.001.

We used single-cell sequence information to assess the connection among FAM110B and 14 functional states in order to continue investigating the possible role of FAM110B in various malignancies (Figure 4A). FAM110B expression and angiogenesis, invasion in PRAD had a positive association. FAM110B was positively associated with angiogenesis, distinction, and inflammation in RB and stemness in AST. Nonetheless, FAM110B was negatively correlated with DNA repair, DNA damage, along with cell cycle in RB (Figures 4B–F). FAM110B was negatively related to DNA repair, DNA damage, and apoptosis in UVM and metastasis, EMT, differentiation, and inflammation in PRAD. In LUAD, FAM110B had a negative relationship with hypoxia, differentiation, and quiescence. The findings demonstrate that FAM110B influences the development of many cancers via several mechanisms.

### 3.5 The connection between FAM110B expression and TME

**FIGURE 5:**
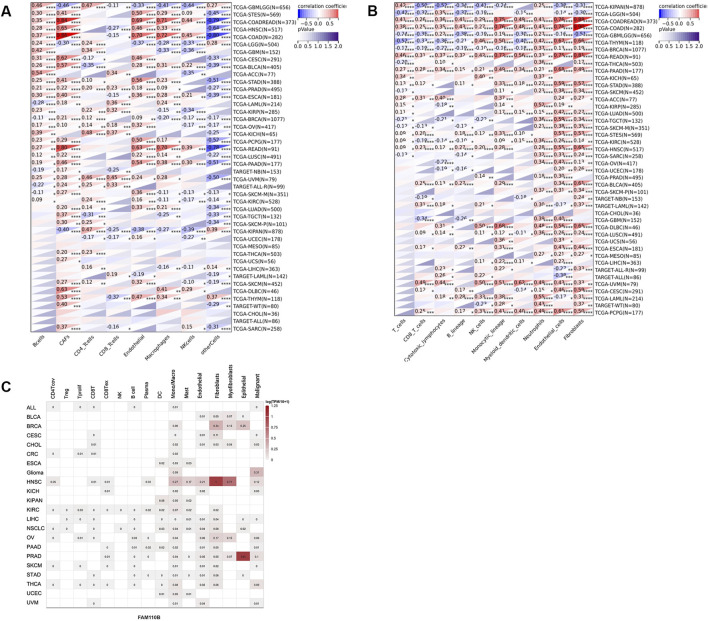
Expression and distribution of FAM110B in TME. **(A,B)** The EPIC **(A)** and MCP-counter **(B)** algorithms are used to analyze the state of immune cell infiltration. **(C)** The TISCH2 website examined the expression of FAM110B in several cell types. *p < 0.05, **p < 0.01, ***p < 0.001.

We subsequently examined it utilizing the QUANTISEQ, EPIC, and MCPCOUNTER methods to evaluate the connection among FAM110B expression and immune cell levels in order to elucidate the connection between FAM110B and immune cell infiltration. The findings showed that the expression of FAM110B considerably influenced the degree of immune cell infiltration across most cancer kinds; the immune cell types most closely associated with FAM110B expression were endothelial cells, neutrophils, monocytes/macrophages, and CAFs (Figures 5A, B; Supplementary Figure S2). We used single-cell sequencing statistical analysis to confirm the aforesaid findings. FAM110B was expressed at higher levels within TME malignant cells, endothelial cells, fibroblasts, and monocytes/macrophages, as Figure 5C illustrates. The results of single-cell data analysis and the results of three classical immune algorithms suggest that immune cell infiltration in the microenvironment of these cancers may be studied more thoroughly. After comparing the outcomes of the three algorithms in more detail, we discovered that FAM110B had a close relationship with a variety of immune cells in KPIAN, COADREAD, KIRC, BRCA, GBMLGG, PAAD, LGG, and HNSC, either exhibiting a substantial positive relationship or a substantial negative relationship. Among them, MCPCOUNTER results showed that in COAD, COADREAD and PAAD, the infiltration levels of ten kinds of immune cells were positively correlated with FAM110B expression, and the correlation coefficient between endothelial cells, fibrocytes, and monocytic cell lines was greater than 0.6. In addition, the expression of FAM110B and the related markers of M1/M2 macrophages, endothelial cells and CAFs were further analyzed. We found that COAD, READ, PAAD, and UVM were positively correlated with the relevant markers of all four cells, and interestingly, the opposite results were observed in LGG (Supplementary Figure S3).

**FIGURE 6:**
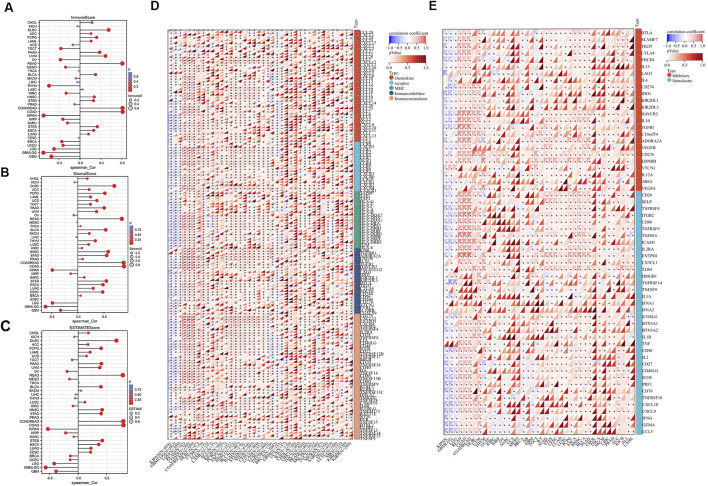
Relationship of FAM110B expression with TME in pan-cancer. **(A–C)** Correlations of FAM110B expression with immune score **(A)**, stromal score **(B)**, and estimated score **(C)** in pan-cancer. **(D)** FAM110B expression and immunological stimulatory and inhibitory gene correlation. **(E)** Correlation of FAM110B expression with immune checkpoint-related genes. *p < 0.05.

Next, we examined the connection between FAM110B expression and TME in pan-cancer. FAM110B expression had a negative correlation with the immune results of GBM, GAMLGG, LGG, UCEC, BRCA, SARC, KIRP, KIPAN, KIRC, THYM, TGCT, along with OV, and had a favorable correlation with the immunological scores for LUAD, ESCA, STES, COAD, COADREAD, STAD, HNSC, BLCA, READ, UVM, PAAD, LAML, PCPG, as well as DLBC (Figure 6A). FAM110B expression had a negative relationship with stromal results for GBM, GBMLGG, LGG, KIRP, and KIPAN and positively associated with CESC, LUAD, ESCA, STES, SARC, COAD, COADREAD, PRAD, STAD, HNSC, LUSC, THYM, SKCM, BLCA, READ, UVM, PAAD, TGCT, UCS, LAML, PCGC, as well as DLBC (Figure 6B). The expression of FAM110B exhibited a negative correlation with the calculated values of GBM, GBMLGG, LGG, BRCA, KIRP, KIPAN, KIRC, and OV. Conversely, the projected values of CESC, LUAD, ESCA, STES, COAD, COADREAD, STAD, HNSC, LUSC, BLCA, READ, UVM, PAAD, LAML, PCPG, and DLBC showed a positive correlation with FAM110B expression (Figure 6C). Immunomodulatory genes encode immunomodulatory proteins that affect the strength and direction of immune responses (Zhao et al., 2022). Tumor cells also inhibit the activity of immune cells by expressing immune regulatory genes, promoting immune escape, and thus resisting the attack of immune cells. To this end, we looked into the connection among FAM110B and immune-related genes in 33 tumors (Figure 6D). FAM110B was discovered to have a substantial positive correlation with the majority of immune-related genes from a pan-cancer standpoint; however, in KIPAN, GBMLGG, and LGG, FAM110B had a negative correlation with the majority of immune-related genes. Furthermore, we discovered that in a large number of malignancies, FAM110B had a positive connection with immunological checkpoints such CD276, EDNRB, CD28, and TLR4 (Figure 6E). To put it briefly, the contribution of abnormal FAM110B expression to TME is not negligible.

**FIGURE 7:**
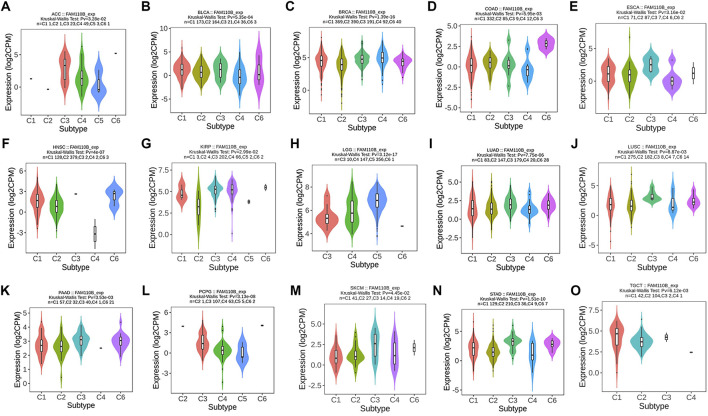
Correlations between FAM110B expression and immune subtypes in pan-cancer. **(A)** ACC. **(B)** BLCA. **(C)** BRCA. **(D)** COAD. **(E)** ESCA. **(F)** HNSC. **(G)** KIRP. **(H)** LGG. **(I)** LUAD. **(J)** LUSC. **(K)** PAAD. (**L**) PCPG. (**M**) SKCM. (**N**) STAD. (**O**) TGCT. C1, wound healing; C2, IFN-gamma dominant; C3, inflammatory; C4, lymphocyte depleted; C5, immunologically quiet; C6, TGF-β dominant.

Certain immune cell subtypes, such as depleted T cells, exhibit specific phenotypes in the tumor microenvironment that are primarily influenced by the characteristics of the tumor cells rather than the organ microenvironment (Liu Y. et al., 2022). Therefore, we finally analyzed the expression of FAM110B in 33 tumors and its correlation with 6 immune subtypes (C1: wound healing, C2: IFN-gamma response, C3: inflammatory, C4: lymphocyte depleted, C5: immunologically quiet, and C6: TGF-β dominant). There were significant differences in different immune subtypes of FAM110B in 15 tumors, including ACC, BLCA, BRCA, COAD, ESCA, HNSC, KIRP, LGG, LUAD, LUSC, PAAS, PCPG, SKCM, STAD, and TGCT (Figures 7A–O).

### 3.6 Forecasting FAM110B-associated tumor immunotherapy outcomes and medication

**FIGURE 8:**
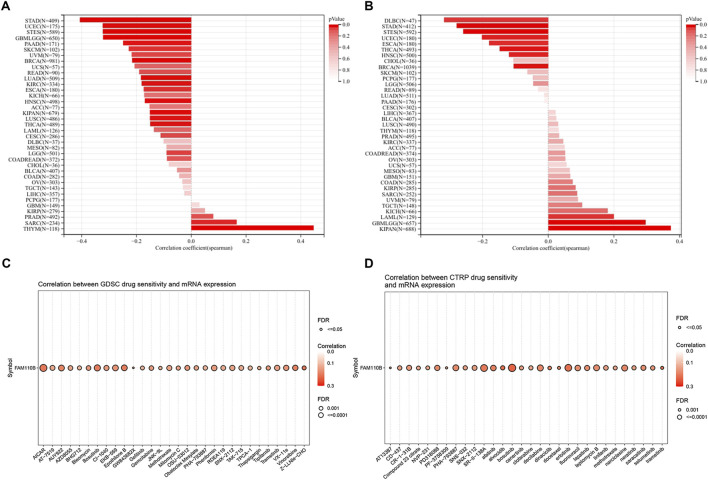
Immunotherapy response and drug-sensitive prediction of FAM110B in pan-cancer. **(A,B)** FAM110B expression and TMB **(A)** and MSI **(B)** correlation in pan-cancer. **(C,D)** The linked medications that target FAM110B were predicted using the GDSC **(C)** and CTRP **(D)** databases. *p < 0.05, **p < 0.01, ***p < 0.001.

TMB and MSI are considered promising biomarkers for predicting immunotherapy efficacy, and patients respond better to immunotherapy when they have high TMB along with MSI (Condelli et al., 2021; Rizzo et al., 2021). In a pan-cancer study, we looked at and contrasted the relationship within FAM110B expression, TMB and MSI. Spearman’s correlational study demonstrated that FAM110B expression within SARC, and THYM had a positive relationship with their TMB, while it had a negative correlation with TMB data in GBMLGG, LGG, LUAD, BRCA, ESCA, STES, KIPAN, STAD, UCEC, HNSC, KIRC, LUSC, THCA, PAAD, as well as SKCM (Figure 8A). Furthermore, FAM110B expression levels in GBMLGG, LAML, and KIPAN showed a positive correlation with MSI. In contrast, it had a negative correlation with MSI in BRCA, ESCA, STES, STAD, UCEC, HNSC, THCA, as well as DLBC (Figure 8B). In addition, when we evaluated FAM110B to widely used indicator of immunotherapy reaction, we discovered that seven immunotherapy groups had an AUC larger than 0.5 (Supplementary Figure S4). Finally, we examined the drug sensitivity of FAM110B expression based on GDSC and CTRP data sets. Drug IC50 examination of FAM110B using the GDSC dataset showed that Z-LLNle-CHO (a γ-Secretase Inhibitor I), AICAR (an activator of AMP-activated protein kinase), and GW843682X (a selective, ATP-competitive inhibitor of PLK1 and PLK3) were the top three medications connected favorably with FAM110B expression (Figure 8C; Supplementary Table S1). The relationship between FAM110B levels and drug sensitivity depending on the CTRP dataset demonstrated that Bosutinib (a tyrosine kinase inhibitor, TKI), alvocidib (an ATP-competitive, Cyclin-dependent kinase inhibitor), and trametinib (a MEK1/2 inhibitor) were the top three medications connected favorably with FAM110B expression (Figure 8D; Supplementary Table S2). As a result, we think that the aberrant expression of FAM110B may somewhat influence the immunotherapy’s effectiveness.

## 4 Discussion

FAM110B is localized in the mitotic spindle and is involved in microtubule nucleation and organization in tissues (Hauge et al., 2007). Ectopic expression of FAM110B can affect the cell cycle progression of G1 phase, which is also one of the characteristics of cancer cells (Schwartz and Shah, 2005). The function of FAM110B in malignant tumors was debatable in the earlier research. Vainio et al. proposed on the basis of genome-wide DNA and RNA information that FAM110B may contribute to the development of castration-resistant prostate cancer (Vainio et al., 2012). However, our previous study used WGCNA analysis, combined with a survival analysis curve and the GEPIA database for validation, and discovered that FAM110B was linked to a good survival time for patients with pancreatic cancer (Li et al., 2023), which was consistent with the results of Xi and Zhang (2018). The FAM110B expression profile, genetic alteration, DNA methylation, RNA modification, clinical importance, and prognostic value in pan-cancer were all systematically analyzed in this work. Additional analyses were conducted to examine the relationships across FAM110B expression and TME, immune subtype, TMB, MSI, and small-molecule medication prediction.

According to our results, most cancer tissues express abnormal expression of FAM110B mRNA compared to normal tissues. The abnormal expression of FAM110B may be influenced by multiple factors and are unable to clarified through genetic alterations, CNA, DNA methylation, and RNA modification alone, which will require deeper exploration in the future. Additionally, clinicopathological stage and histological grade were correlated with FAM110B mRNA expression levels in some tumors. Kaplan-Meier method and COX regression analysis indicated that FAM110B may be a potential prognostic biomarker for a variety of cancers. Through the above analysis, it was found that FAM110B played a prominent role in PAAD. In this study, the increased expression of FAM110B was closely related to the histological grade, clinical stage and prognosis of PAAD. We further carried out a univariate logistic analysis on the expression level of FAM110B and clinicopathological features, and the results showed that FAM110B was only correlated with histological grade and pathological T-stage, and had no statistical significance with age and gender (Supplementary Table S3). We further constructed a prognostic risk scoring system by univariate and multivariate COX proportional risk regression, and the results showed that FAM110B was an independent marker of OS prognosis in PAAD patients (Supplementary Table S4). Additionally, single-cell sequencing data showed that FAM110B expression was linked to the cell cycle, distinction, angiogenesis, damaged DNA, hypoxia, inflammation, and stemness in the majority of cancers, according to single-cell sequencing information. Xie et al. (2020) showed that overexpressed FAM110B could affect the progression of G1 phase of the cell cycle, and was found to limit the proliferation and invasion of NSCLC by inhibiting Wnt/β-related protein signaling. Vainio et al. (2012) demonstrated that downregulation of FAM110B decreased the expression of genes associated with DNA repair and cell cycle G2/M transition in PRAD cells. At present, there are still huge gaps in the research of FAM110B in cancer function. In this study, FAM110B was analyzed for the first time in 14 functional states in multiple cancers. However, more *in vitro* experiments are needed to further validate our results.

The intricate network of cells, growth variables, and signaling compounds that controls tumor growth and immune evasion is known as the tumor microenvironment (TME) (Sahoo et al., 2018; McCoach and Bivona, 2019). A large amount of evidence shows that TME is of great value in predicting prognosis and evaluating therapeutic effect factors (Binnewies et al., 2018). Previous studies have shown that FAM110B is also involved in the regulation of antigen presentation on the surface of prostate cancer cells, thus allowing the immune escape of tumor cells (Vainio et al., 2012). In addition, FAM110B also acts as an important hub gene in the immune microenvironment of colon cancer cells, and its expression level is negatively correlated with tumor purity and positively correlated with the infiltration of CD4 T cells, macrophages, neutrophils and dendritic cells. (Wang et al., 2020). However, the relationship between FAM110B and pan-cancer TME and tumor immune cell infiltration remains largely unknown. In this study, EPIC and MCPCOUNTER algorithms were used to find important associations between FAM110B expression and TME endothelial cells, neutrophils, mononuclear/macrophages, and CAFs, suggesting that elevated FAM110B expression may encourage tumor cells to create an environment that is conducive to growth. Stromal and immune cells, as two non-tumor parts of the TME, have been suggested to be beneficial for the diagnosis and prognostic evaluation of tumors (Yoshihara et al., 2013). As a result, we used immune scores, stromal scores, and ESTIMATE to assess the purity of immune cells, stromal cells, and tumor within TME. It appears that FAM110B interacted with immune and tumor cells in tumors because we observed that FAM110B had a positive correlation with a stromal and immune score within 13 malignancies and a negative correlation with a score in 5 cancers. Furthermore, our study showed a strong correlation between immune regulatory genes and FAM110B. In summary, the expression of FAM110B affects the composition of TME.

At present, as one of the main methods of immunotherapy, immune checkpoint inhibitor therapy has become the focus of many studies and clinical trials (Soularue et al., 2018; Li et al., 2019). In this study, the expression of FAM110B was positively correlated with various immune checkpoints such as PDCD1, CTLA4, EDNRB, TLR4, etc., indicating that FAM110B may be a new target for tumor immunotherapy. However, different immune subtypes may have different effects on immune checkpoint inhibitors. For example, a tumor microenvironment rich in effector T cells (specifically CD8+ T cells) often predicts a good response to immune checkpoint inhibitor therapy (Petralia et al., 2024). We further analyzed the relationship between FAM110B and immune subtypes in order to provide more individualized immunotherapy guidance for patients. TMB and MSI are effective biological indicators to predict the effect of tumor immunotherapy, and tumors with high TMB and MSI are more sensitive to immunotherapy (Darragh et al., 2018; Reck et al., 2019; Filipovic et al., 2020). However, assessing TMB in routine clinical practice is challenging due to high sequencing costs and long turnaround times. Fascinatingly, our results showed that FAM110B expression was negatively correlated with TMB and MSI within BRCA, ESCA, STAD, STES, HNSC, and THCA. Therefore, we conjectured that decreased expression of FAM110B in these six tumors would offer a better improvement in survival after immunotherapy. We also looked into the connection between FAM110B mRNA and anti-cancer drug sensitivity using the GDSC database, which will help with patient prediction and clinical therapy selection. Based on these findings, the potential mechanism of FAM110B regulation of TME is expected to be revealed from more perspectives in the future, and it is also conducive to providing a theoretical basis for personalized treatment of cancer immunotherapy.

This study elucidated the role of FAM110B in the occurrence of various tumors from multiple perspectives, and provided a certain basis for further research on the specific mechanism of FAM110B in cancer progression and treatment. Admittedly, there are some limitations to our study that should not be ignored. First off, all of the results were obtained through bioinformatic analyses based on large datasets. Future work is needed to further explore the specific molecular mechanisms of FAM110B in cell cycle regulation, tumor microenvironment (TME) regulation, and immune escape through *in vitro* experiments and *in vivo* models. In addition to genome-level analysis, future studies should also be extended to the proteomic level to assess FAM110B protein expression levels, post-translational modifications, and interactions with other proteins.

## 5 Conclusion

In conclusion, this study systematically analyzed FAM110B using bioinformatics methods, revealing the important role of FAM110B in multiple tumor prognosis and immune invasion, as well as the correlation between tumor microenvironment, which may provide new individualized strategies for clinical immunotherapy.

## Data Availability

The original contributions presented in the study are included in the article/Supplementary Material, further inquiries can be directed to the corresponding author.
