# Supplementary material for: Pan-cancer analysis and single-cell analysis reveals FAM110B as a potential target for survival and immunotherapy
Source: Front Mol Biosci. 2024 Aug 7;11:1424104. doi: 10.3389/fmolb.2024.1424104 (PMC11335499; doi:10.3389/fmolb.2024.1424104)
Supplement: Supplementary file 1 [file DataSheet2.DOCX]

Supplementary methods

1. **FAM110B Expression and Datasets Obtained:** we extracted the expression data of ENSG00000169122 (FAM110B) gene in each sample, and further screened the sample sources as follows: Solid Tissue Normal, Primary Solid Tumor, Primary Tumor, Normal Tissue, Primary Blood Derived Cancer - Bone Marrow, Primary Blood Derived Cancer-Peripheral Blood samples. After log2(x+0.001) transformation was performed for each expression value, the cancer species with less than 3 samples were eliminated, and the expression data of 34 cancer species were finally obtained.
2. **Datasets used for Tumor Immune Single-cell Hub 2 (TISCH2,** [**http://tisch.comp-genomics.org/home/**](http://tisch.comp-genomics.org/home/) **):** ALL-GSE132509, BLCA-GSE130001, BRCA-GSE138536, CESC-GSE168652, CHOLGSE142784, CRC-GSE136394, ESCA-GSE154763, Glioma-GSE84465, HNSC-GSE103322, KICH-GSE159115, KIPAN-GSE154763, KIRC-GSE111360, LIHC-GSE166635, NSCLC-GSE146100, OV-GSE147062, PAAD-CRA001160, PRAD-GSE137829, SKCM-GSE72056, STAD-GSE167297, THCA-GSE148673, UCEC-GSE154763, and UVM-GSE138433. Single-cell sequence data of FAM110B gene was analyzed to clarify the expression level of FAM110B in each cell type.
3. **Analysis of immune checkpoint pathway genes and immune regulatory genes:** Obtain data on 60 immune checkpoint pathway genes (including inhibitory (24) and stimulatory (36)) and 150 immune regulatory genes (including chemokine (41), receptor (18), MHC (21), immunoinhibitor (24), immunostimulator (46)) in each sample. Then, all normal samples were filtered, and log2(x+0.001) transformation was further carried out for each expression value. Spearman correlation was used to analyze the expression relationship between FAM110B and immune checkpoint genes and immune regulatory genes, and Heatmap was used for visualization.
4. **Quality control of CancerSEA:** First, in order to ensure that all single cells are cancer cells and not mixed with normal cells in the tumor microenvironment, non-malignant single cells are removed according to the following criteria: (i) if the original papers have included the information about whether cells are malignant or not, only the malignant cells were remained; (ii) using an RNAseq-inferred copy number variation (CNV) approach to distinguish malignant cells from non-malignant cells. After that, 68 708 malignant single cells remained. Second, two quality measures were calculated for each cell, including the number of genes with detectable expression (i.e. expression levels are greater than 0) and the average expression level of 87 housekeeping genes collected in. We then excluded cells with the number of expressed genes fewer than 1000 or with the average housekeeping expression <2.
5. **Quality control of TISCH:** Cell number per dataset (>1000)

UMI count per cell (>1000)

Gene number per cell (>500)
