# Supplementary material for: Pan-cancer analysis and single-cell analysis reveals FAM110B as a potential target for survival and immunotherapy
Source: Front Mol Biosci. 2024 Aug 7;11:1424104. doi: 10.3389/fmolb.2024.1424104 (PMC11335499; doi:10.3389/fmolb.2024.1424104)
Supplement: Supplementary file 2 [file DataSheet1.docx]

**
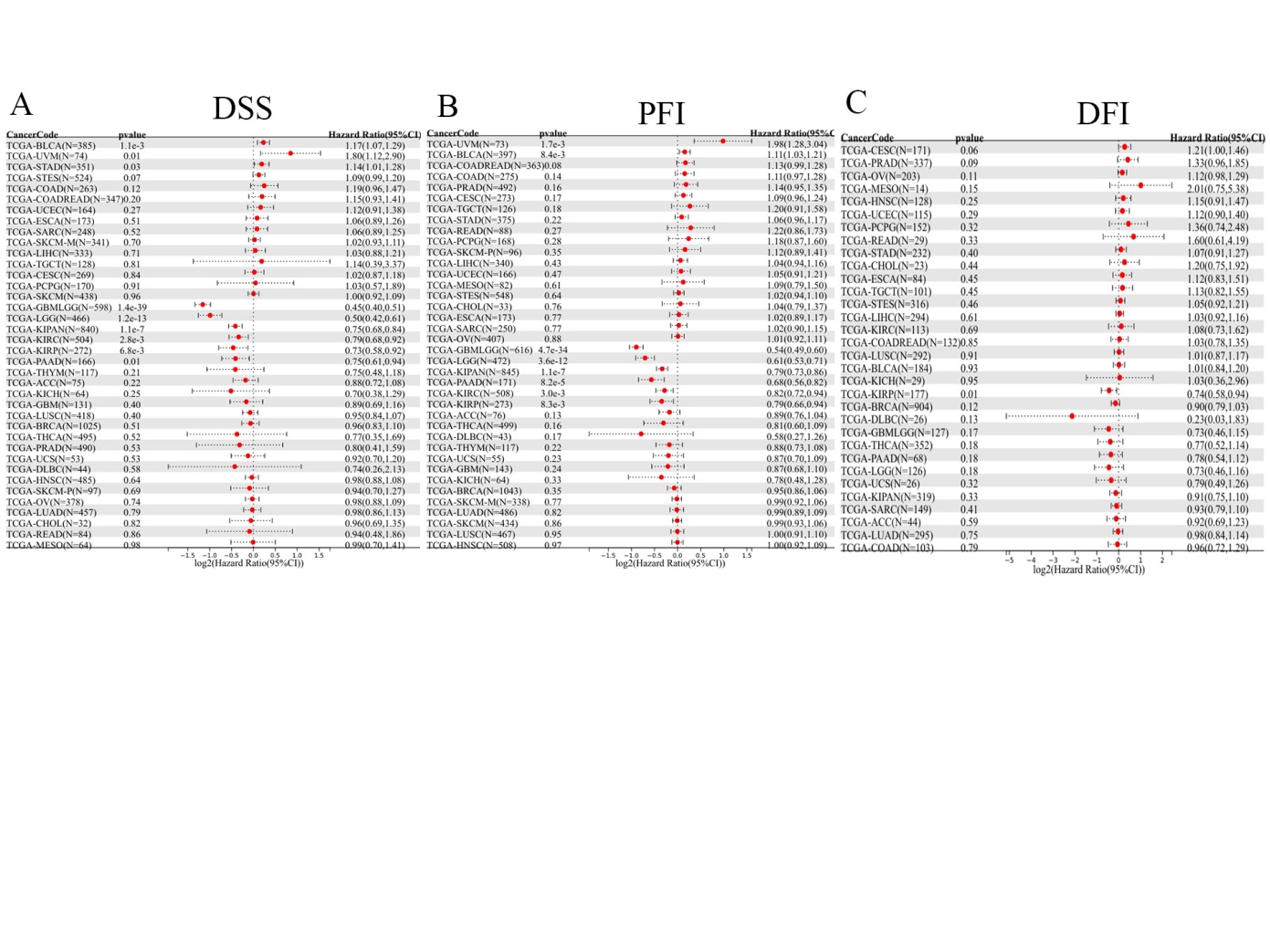
**

**Supplementary Figure 1.** Effect of FAM110B expression on prognosis of pan-cancer. (A-C) Cox proportional hazards regression models were used to evaluate the association of FAM110B expression with disease-specific survival (DSS) (A), progression-free interval (PFI) (B), and disease-free interval (DFI) (C).


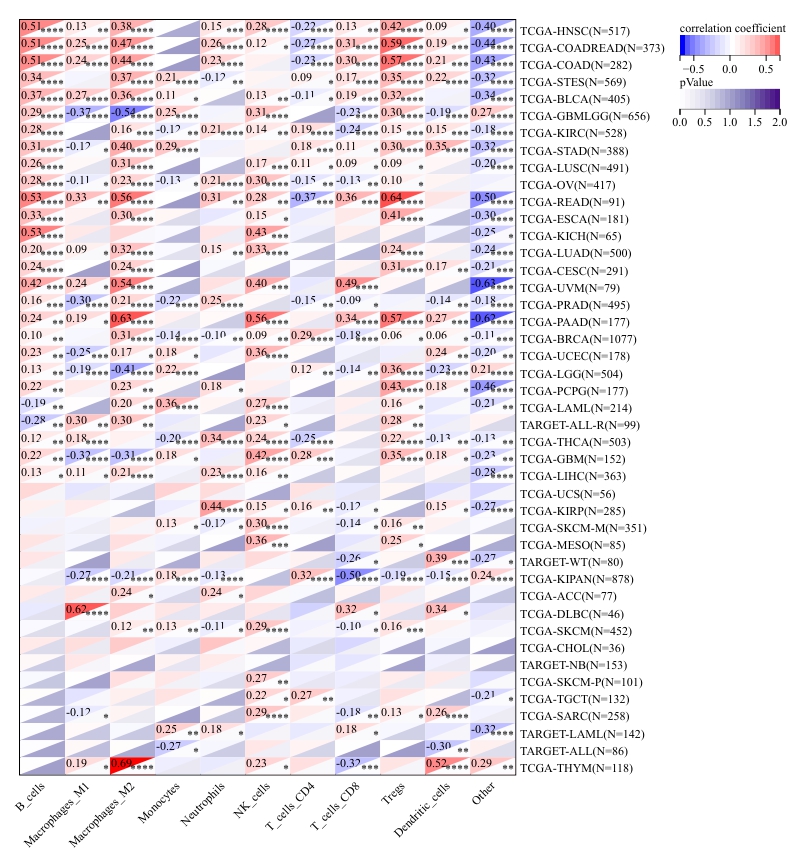


**Supplementary Figure 2.** Correlation of FAM110B with the level of immune infiltrating cells. FAM110B was closely related to the immune infiltration level in cancers analyzed via QUANTISEQ algorithms. *p < 0.05; **p < 0.01; ***p < 0.001; ****p < 0.0001.


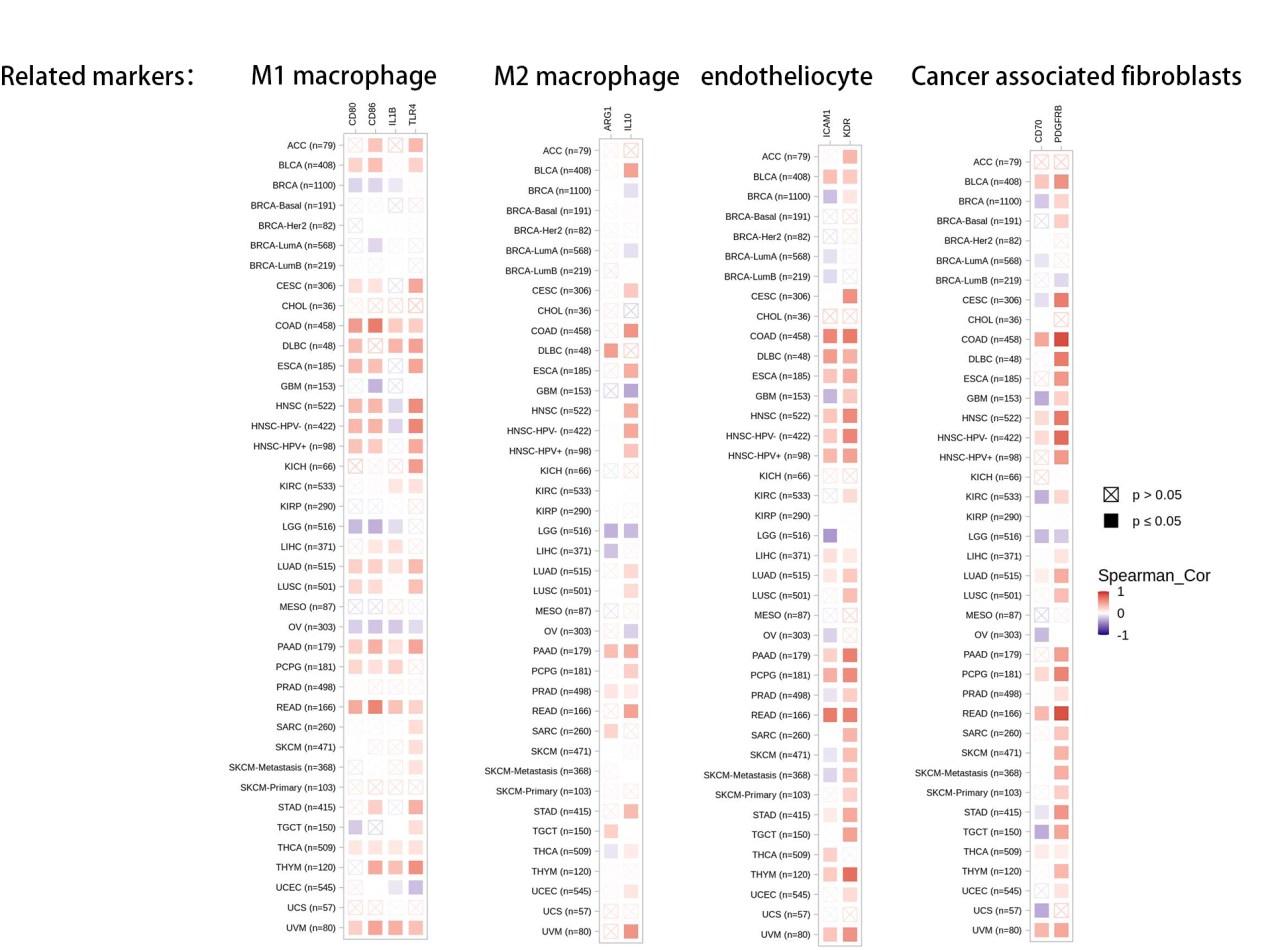


**Supplementary Figure 3. The associated markers of FAM110B and immune cells were analyzed in the TIMER database.** Pan-cancer analysis of FAM110B expression and M1/M2, endothelial cells, CAFs cell markers.


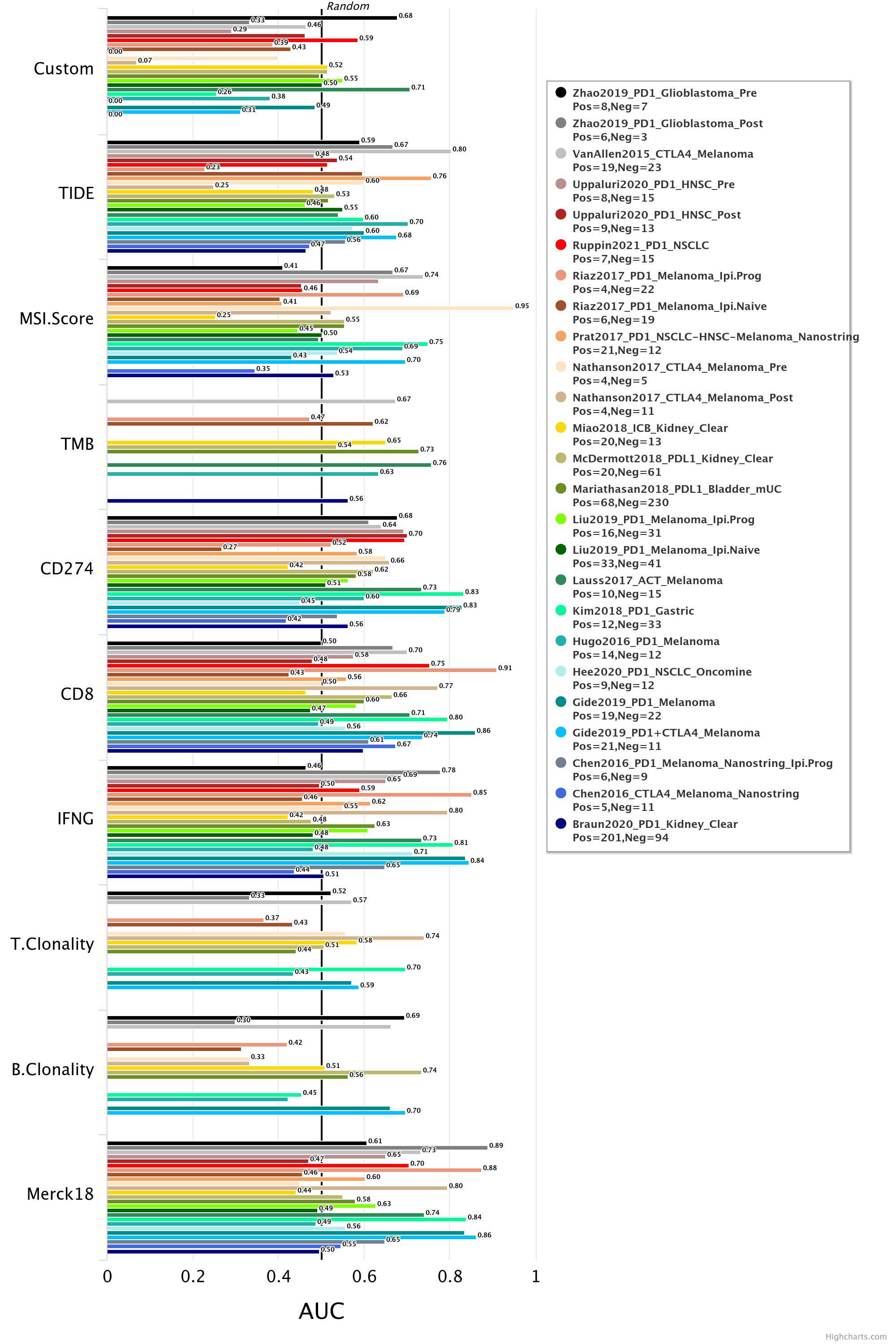


**Supplementary Figure 4.** Biomarker comparison of FAM110B in the immunotherapy group.

| **Supplementary Table 1. Table summarizes the correlation between gene expression and the sensitivity of GDSC drugs (top 30) in pan-cancer** | | | |
| --- | --- | --- | --- |
| symbol | drug | Pearson correlation | False discovery rate |
| FAM110B | Z-LLNle-CHO | 0.20871891 | 0.000592822 |
| FAM110B | AICAR | 0.204145966 | 3.50198E-08 |
| FAM110B | GW843682X | 0.198432752 | 0.006603744 |
| FAM110B | Epothilone B | 0.198194448 | 4.22418E-06 |
| FAM110B | Bosutinib | 0.198188491 | 1.32108E-06 |
| FAM110B | Vinorelbine | 0.193113276 | 0.00001715 |
| FAM110B | Paclitaxel | 0.189464333 | 0.014999319 |
| FAM110B | AUY922 | 0.187456958 | 3.80693E-06 |
| FAM110B | S-Trityl-L-cysteine | 0.182739173 | 0.009080207 |
| FAM110B | BI-2536 | 0.180607399 | 0.033775568 |
| FAM110B | JW-7-52-1 | 0.176502403 | 0.015399243 |
| FAM110B | CGP-60474 | 0.168551549 | 0.014073384 |
| FAM110B | VX-11e | 0.168388658 | 0.000022141 |
| FAM110B | EKB-569 | 0.167443944 | 4.93968E-06 |
| FAM110B | Sunitinib | 0.167083809 | 0.008492927 |
| FAM110B | OSU-03012 | 0.165628234 | 0.000069951 |
| FAM110B | Bleomycin | 0.162959605 | 0.000144029 |
| FAM110B | A-770041 | 0.162853122 | 0.01592958 |
| FAM110B | Tipifarnib | 0.161459156 | 0.000371674 |
| FAM110B | CGP-082996 | 0.161389938 | 0.025912299 |
| FAM110B | CI-1040 | 0.159069261 | 0.000041562 |
| FAM110B | Gemcitabine | 0.157988912 | 0.000168485 |
| FAM110B | Phenformin | 0.157505412 | 0.000010568 |
| FAM110B | VX-680 | 0.156268198 | 0.042275053 |
| FAM110B | Trametinib | 0.150916562 | 0.000022665 |
| FAM110B | Mitomycin C | 0.150705208 | 0.000439111 |
| FAM110B | Obatoclax Mesylate | 0.14879475 | 0.00023537 |
| FAM110B | AZ628 | 0.147648135 | 0.023702889 |
| FAM110B | SNX-2112 | 0.147534118 | 0.000028213 |
| FAM110B | Methotrexate | 0.147476212 | 0.000045305 |

| **Supplementary Table 2. Table summarizes the correlation between gene expression and the sensitivity of CTRP drugs (top 30) in pan-cancer** | | | |
| --- | --- | --- | --- |
| symbol | drug | Pearson correlation | False discovery rate |
| FAM110B | bosutinib | 0.230629222 | 3.60668E-09 |
| FAM110B | alvocidib | 0.217987543 | 0.000100249 |
| FAM110B | trametinib | 0.216087403 | 0.000688154 |
| FAM110B | erlotinib | 0.214638529 | 4.34886E-08 |
| FAM110B | SR-II-138A | 0.201777375 | 2.9632E-08 |
| FAM110B | dinaciclib | 0.197914353 | 0.000341817 |
| FAM110B | docetaxel | 0.195551371 | 0.000692983 |
| FAM110B | afatinib | 0.195132868 | 3.06889E-06 |
| FAM110B | narciclasine | 0.18614773 | 8.57076E-07 |
| FAM110B | decitabine | 0.184294288 | 6.40051E-07 |
| FAM110B | AT13387 | 0.183465146 | 0.001330178 |
| FAM110B | PD318088 | 0.182491806 | 8.12741E-06 |
| FAM110B | PHA-793887 | 0.18015666 | 1.19263E-06 |
| FAM110B | lapatinib | 0.179053121 | 0.0000115 |
| FAM110B | PF-3758309 | 0.178620363 | 0.001217666 |
| FAM110B | leptomycin B | 0.177815511 | 1.26237E-06 |
| FAM110B | saracatinib | 0.17762582 | 0.000014383 |
| FAM110B | fluorouracil | 0.176264451 | 3.3548E-06 |
| FAM110B | selumetinib | 0.167306576 | 0.000075858 |
| FAM110B | tivantinib | 0.167184184 | 0.002849096 |
| FAM110B | CR-1-31B | 0.161181062 | 0.000013479 |
| FAM110B | canertinib | 0.159665763 | 0.000140656 |
| FAM110B | bleomycin A2 | 0.15840822 | 0.00076436 |
| FAM110B | SNX-2112 | 0.158192989 | 0.000020289 |
| FAM110B | SNS-032 | 0.15669659 | 0.000097565 |
| FAM110B | neratinib | 0.156246242 | 0.000107037 |
| FAM110B | linifanib | 0.156122462 | 0.000040658 |
| FAM110B | methotrexate | 0.150958831 | 0.000180606 |
| FAM110B | CD-437 | 0.149847912 | 0.000077788 |
| FAM110B | COL-3 | 0.149246323 | 0.000943229 |

**Supplementary Table 3. Univariate logistic analysis of FAM110B expression and clinicopathological features in PAAD.**

| Characteristics | Total (N) | OR (95% CI) | P value |
| --- | --- | --- | --- |
| Pathologic T stage (T3&T4 vs. T1&T2) | 177 | 0.404 (0.178 – 0.917) | **0.030** |
| Pathologic N stage (N1 vs. N0) | 174 | 0.968 (0.502 – 1.867) | 0.923 |
| Pathologic M stage (M1 vs. M0) | 85 | 1.291 (0.205 – 8.146) | 0.786 |
| Gender (Male vs. Female) | 179 | 0.710 (0.393 – 1.283) | 0.257 |
| Age (> 65 vs. <= 65) | 179 | 0.782 (0.435 – 1.408) | 0.413 |
| Histologic grade (G3&G4 vs. G1&G2) | 177 | 0.499 (0.255 – 0.975) | **0.042** |
| Pathologic stage (Stage IV&Stage III vs. Stage I&Stage II) | 176 | 1.024 (0.248 – 4.230) | 0.974 |

**Supplementary Table 4. Univariate and multivariate COX regression analysis of FAM110B and PAAD patients prognosis**

| Characteristics | Total(N) | Univariate analysis | |  | Multivariate analysis | |
| --- | --- | --- | --- | --- | --- | --- |
|  |  | Hazard ratio (95% CI) | P value |  | Hazard ratio (95% CI) | P value |
| FAM110B | 179 |  |  |  |  |  |
| Low | 89 | Reference |  |  | Reference |  |
| High | 90 | 0.573 (0.376 - 0.874) | **0.010** |  | 0.573 (0.376 - 0.874) | **0.010** |
| Gender | 179 |  |  |  |  |  |
| Female | 80 | Reference |  |  |  |  |
| Male | 99 | 0.813 (0.541 - 1.222) | 0.319 |  |  |  |
| Race | 175 |  |  |  |  |  |
| Asian | 11 | Reference |  |  |  |  |
| Black or African American | 6 | 1.210 (0.324 - 4.519) | 0.777 |  |  |  |
| White | 158 | 1.264 (0.511 - 3.127) | 0.613 |  |  |  |
| Histologic grade | 177 |  |  |  |  |  |
| G1&G2 | 127 | Reference |  |  |  |  |
| G4&G3 | 50 | 1.532 (0.993 - 2.363) | 0.054 |  |  |  |
| Age | 179 |  |  |  |  |  |
| <= 65 | 94 | Reference |  |  |  |  |
| > 65 | 85 | 1.285 (0.853 - 1.937) | 0.230 |  |  |  |
| Pathologic stage | 176 |  |  |  |  |  |
| Stage I&Stage II | 168 | Reference |  |  |  |  |
| Stage III&Stage IV | 8 | 0.676 (0.213 - 2.145) | 0.507 |  |  |  |
